# Robot-Assisted Radical Prostatectomy Beyond the Learning Curve: Does Prior Laparoscopic Experience Influence Surgical Outcomes?

**DOI:** 10.3390/cancers18040548

**Published:** 2026-02-07

**Authors:** Alberto Zambudio-Munuera, Irene Millán-Ramos, Patricia Rodríguez-Parras, Francisco Gutiérrez-Tejero, María Teresa Melgarejo-Segura, Miguel Arrabal-Martin, Miguel Ángel Arrabal-Polo

**Affiliations:** 1 Department of Urology, San Cecilio Clinical University Hospital of Granada, Av. del Conocimiento, s/n, 18007 Granada, Spain; alberto.zambudio.sspa@juntadeandalucia.es (A.Z.-M.); irene.millan.sspa@juntadeandalucia.es (I.M.-R.); patricia.rodriguez.parras.sspa@juntadeandalucia.es (P.R.-P.); francisco.gutierrez.tejero.sspa@juntadeandalucia.es (F.G.-T.); mariat.melgarejo.sspa@juntadeandalucia.es (M.T.M.-S.); miguel.arrabal.sspa@juntadeandalucia.es (M.A.-M.); 2 Granada Biosanitary Research Institute (ibs.GRANADA), 18012 Granada, Spain

**Keywords:** learning curve, prostatectomy, prostatic neoplasms, robotic surgical procedures, treatment outcome

## Abstract

**Background/Objectives**: Robot-assisted radical prostatectomy (RARP) is widely used in contemporary prostate cancer surgery; however, surgeons enter robotic practice through heterogeneous training pathways. This study aimed to compare early oncological and functional outcomes after RARP between two experienced robotic surgeons with different surgical backgrounds after completion of the learning curve. **Methods**: We conducted a retrospective, consecutive, single-center study including patients undergoing RARP after completion of the learning curve (> 40 cases) by two experienced robotic surgeons with different surgical backgrounds. Baseline characteristics, perioperative variables, and early oncological and functional outcomes were compared between surgeons. Pentafecta achievement was assessed as an exploratory composite outcome. Appropriate non-parametric and categorical statistical tests were applied as appropriate. **Results**: Ninety-three patients were included (55 operated on by surgeon A and 38 by surgeon B). Preoperative clinical and pathological characteristics were largely comparable between groups, except for prostate volume. Median operative time was significantly shorter for surgeon A (70 vs. 120 min, *p* < 0.001). Postoperative morbidity was low, with no major complications and no differences in length of hospital stay. At 6 months, urinary continence and erectile function recovery rates were high and comparable between surgeons. Oncological outcomes, including positive surgical margin rates and biochemical recurrence, did not differ significantly, although recurrence events were infrequent and follow-up was limited. Overall pentafecta achievement was modest and similar between groups (23.6% vs. 21.1%, *p* = 0.77), with positive surgical margins emerging as the main limiting factor. **Conclusions**: In this exploratory post-learning curve analysis, early oncological and functional outcomes after RARP were similar between surgeons with different surgical backgrounds. These findings should be interpreted cautiously and considered hypothesis-generating.

## Simple Summary

Robot-assisted radical prostatectomy is increasingly performed by surgeons with heterogeneous training backgrounds. In recent years, a growing number of surgeons are being incorporated directly into robotic programs without prior laparoscopic experience, raising questions about the relevance of previous surgical background once robotic proficiency is achieved. Whether prior laparoscopic training influences patient outcomes after completion of the robotic learning curve remains unclear. In this retrospective study, we compared early oncological and functional outcomes after robot-assisted radical prostatectomy between two experienced robotic surgeons with different surgical backgrounds, analyzing only procedures performed after the learning curve had been surpassed. Early functional recovery and oncological outcomes were similar between surgeons, while overall pentafecta achievement was limited in both groups. Failure to achieve pentafecta was mainly driven by positive surgical margins rather than functional outcomes. These findings suggest that, once robotic proficiency is achieved, early results may be more influenced by tumor characteristics and surgical strategy than by previous laparoscopic experience. However, given the exploratory nature of the study and limited follow-up, the results should be interpreted with caution.

## 1. Introduction

Robot-assisted radical prostatectomy (RARP) has become the predominant surgical approach for both localized and locally advanced prostate cancer [1,2], supported by robust evidence demonstrating favorable perioperative, oncological, and functional outcomes [3,4]. Robotic platforms provide a high degree of standardization, yet surgical outcomes are not independent of the operator. Individual factors, especially the surgeon’s experience and specific training, remain influential. Consequently, many studies have investigated the RARP learning curve. These reports consistently show that operative efficiency and key quality metrics improve as the surgeon’s procedural volume increases [5].

Prior laparoscopic experience has traditionally been regarded as beneficial when transitioning to robotic platforms [6]. However, RARP represents a distinct surgical paradigm rather than a direct extension of conventional laparoscopy. Differences in visuomotor coordination, console-based instrument control, and the absence of tactile feedback indicate that skill transfer from laparoscopy to robotics may be incomplete. Moreover, robotic platforms overcome several intrinsic technical and ergonomic limitations of conventional laparoscopy—such as restricted range of motion, tremor amplification, and surgeon fatigue—which may attenuate the impact of prior laparoscopic experience once robotic proficiency is achieved [7]. As a result, the influence of prior laparoscopic training on long-term surgical performance after completion of the robotic learning curve remains uncertain. Recent studies have suggested that oncological outcomes, including BCR, may vary among surgeons despite comparable procedural volumes [8].

Most RARP research focuses on the early stages of robotic training rather than long-term expertise. Unfortunately, these studies often conflate initial learning curve effects with the surgeon’s sustained performance over time [5,6,9,10]. Consequently, it is still uncertain whether differences between surgeons with and without prior laparoscopic experience persist after the learning curve has been completed and technical standardization achieved. This question has gained increasing relevance in current training pathways, where direct transition to robotic surgery without extensive laparoscopic exposure is becoming more common.

Composite outcome measures, such as the pentafecta, have been proposed to provide a comprehensive assessment of surgical quality after RARP by integrating oncological control, functional recovery, and perioperative safety into a single endpoint [11,12]. Evaluating surgical performance after completion of the learning curve using standardized composite outcomes may therefore offer a more meaningful comparison of proficiency between surgeons with differing training backgrounds.

The present study aimed to compare perioperative, functional, and early oncological outcomes of robot-assisted radical prostatectomy performed by two experienced robotic surgeons with different surgical backgrounds at a single institution, after completion of the learning curve. In addition, pentafecta achievement was explored as a composite outcome to provide an integrated assessment of surgical performance and to identify the factors most strongly limiting its attainment in a post-learning curve setting.

## 2. Materials and Methods

### 2.1. Study Design and Population

A retrospective, single-center observational study was conducted including consecutive patients who underwent RARP at San Cecilio University Hospital (Granada, Spain) between January 2024 and May 2025.

All procedures were performed by two dedicated robotic surgeons working contemporaneously within the same institutional setting. Surgeon B had prior experience in laparoscopic prostate surgery, whereas Surgeon A was trained directly in robot-assisted surgery without previous laparoscopic experience.

To ensure assessment beyond the initial learning phase, only procedures performed after the first 40 RARP cases by each surgeon were included in the analysis [13,14].

### 2.2. Eligibility Criteria

The study included men with histologically confirmed prostate adenocarcinoma (ISUP Grade Group 1–5) who underwent RARP for pT2–pT3, pN0M0 disease. Patients were excluded in cases of follow-up shorter than 6 months, incomplete clinical or pathological data, unavailable or persistently detectable postoperative PSA values, or prior neoadjuvant or adjuvant radiotherapy or androgen deprivation therapy.

### 2.3. Surgical Technique

All procedures were performed using the da Vinci Xi robotic surgical system (Intuitive Surgical, Sunnyvale, CA, USA) through a standardized transperitoneal approach. The surgical technique followed a predefined stepwise sequence that remained unchanged throughout the study period and was identical for both surgeons.

After robotic docking, the procedure included bladder neck dissection, identification and dissection of the vas deferens and seminal vesicles, posterior dissection, and posterolateral dissection at the level of the neurovascular bundles. Nerve-sparing was attempted bilaterally or unilaterally based on preoperative oncological risk assessment, prioritizing preservation in regions with lower tumor burden and adopting a more conservative approach in areas with suspected higher tumor involvement.

The procedure was completed with anterior and posterior apical dissection, urethral transection, and vesicourethral anastomosis using a double-armed barbed suture according to the Van Velthoven technique. No technical modifications in anastomotic reconstruction or nerve-sparing strategy were introduced during the study period.

In patients with high-grade disease (ISUP 3–5), preoperative staging included PSMA PET imaging. In this subgroup, extended pelvic lymph node dissection was performed according to institutional practice, and all cases showed negative nodal findings. No modifications to the surgical technique were introduced based on lymph node management during the study period.

### 2.4. Data Collection

Data collection encompassed preoperative, intraoperative, and postoperative variables. Preoperative assessment included patient age, preoperative prostate-specific antigen (PSA) levels, biopsy ISUP Grade Group (International Society of Urological Pathology), number of positive biopsy cores, prostate volume, and multiparametric magnetic resonance imaging findings, reported according to the PI-RADS score.

Intraoperative variables comprised operative time, the need for blood transfusion, intraoperative complications, and conversion to open surgery. Estimated blood loss was not analyzed, as its recording was not standardized across cases.

Postoperative evaluation included length of hospital stay, postoperative complications classified according to the Clavien–Dindo system, hospital readmission, vesicourethral anastomotic stricture, functional outcomes, and longitudinal PSA follow-up. Urinary continence was defined as the use of no pads or one safety pad per day, erectile function as an erection hardness score of 3–4, and BCR as two consecutive PSA values ≥0.2 ng/mL. All prostatectomy specimens were evaluated by dedicated genitourinary pathologists according to contemporary pathological reporting standards.

### 2.5. Definition of Pentafecta

The classical pentafecta was analyzed as an exploratory composite outcome and was defined as the simultaneous achievement of the following five criteria: negative surgical margins, absence of biochemical recurrence, urinary continence, preservation of erectile function, and absence of postoperative complications of Clavien–Dindo grade III or higher.

### 2.6. Statistical Analysis

Statistical analyses were performed using IBM SPSS Statistics version 26.0 (IBM Corp., Armonk, NY, USA). Continuous variables were assessed for normality using visual inspection of histograms and the Shapiro–Wilk test and are reported as median with interquartile range (IQR) or mean ± standard deviation, as appropriate. Categorical variables are presented as absolute frequencies and percentages.

Comparisons between surgeons were performed using the Mann–Whitney U test for continuous variables and the chi-square test or Fisher’s exact test for categorical variables, as appropriate.

Oncological and functional outcomes were analyzed descriptively and comparatively according to surgeon. Pentafecta achievement was evaluated as a composite binary outcome and further explored through descriptive analysis of its individual components to identify the main factors limiting its achievement.

No formal sample size calculation was performed, as this was an exploratory analysis based on all eligible consecutive cases available after completion of the learning curve for both surgeons.

All tests were two-sided, and a *p* value < 0.05 was considered statistically significant.

Given the modest sample size and the low number of oncological events, multivariable regression analyses were not performed to avoid overfitting and unstable estimates. Accordingly, the statistical approach was intentionally limited to descriptive and exploratory comparisons.

### 2.7. Use of Artificial Intelligence-Assisted Tools

Artificial intelligence tools, ChatGPT 4.0 (OpenAI, San Francisco, CA, USA) was used solely for language refinement and stylistic editing; all scientific content, data analysis, and interpretations were reviewed and approved by the authors.

### 2.8. Ethical Approval

The study was approved by the Institutional Review Board of San Cecilio University Hospital (Granada, Spain) (approval code: PR001, approval date: 30 September 2025). Due to the retrospective nature of the study, the requirement for informed consent was waived.

## 3. Results

### 3.1. Baseline Patient Characteristics and Follow-Up

A total of 93 patients were included, 55 operated on by Surgeon A and 38 by Surgeon B. Baseline preoperative characteristics according to surgeon are summarized in Table 1.

*p* values were calculated using the Mann–Whitney U test for continuous variables and the chi-square test or Fisher’s exact test for categorical variables, as appropriate. A two-sided *p* value < 0.05 was considered statistically significant.

The two groups were comparable in terms of age, body mass index, preoperative PSA, PSA density, biopsy ISUP grade distribution, and preoperative risk profile, with no statistically significant differences observed between surgeons.

Median prostate volume was significantly higher in patients operated on by surgeon B compared with surgeon A (46.0 vs. 43.0 mL, *p* = 0.025).

Preoperative multiparametric MRI findings showed no statistically significant differences between groups (*p* = 0.076), although a numerically higher proportion of PI-RADS 5 lesions was observed in the cohort treated by surgeon A.

Overall, baseline characteristics indicated a largely comparable preoperative case mix between both surgeons, with prostate volume being the only variable showing a statistically significant difference.

The median follow-up was 11 months in both groups, with no significant difference in follow-up duration between surgeon A and surgeon B.

### 3.2. Intraoperative and Postoperative Outcomes

Intraoperative outcomes are summarized in Table 2. Median operative time was significantly shorter for surgeon A compared with surgeon B (70 (60–100) vs 120 (80–165), *p* < 0.001). No patient in either group required intraoperative blood transfusion or conversion to open surgery.

Postoperative outcomes were comparable between groups. Complications were infrequent and limited to Clavien–Dindo grade I–II events, with no major complications (grade ≥ III) observed. Length of hospital stay was similar between surgeons, with a median of 1 day in both groups (*p* = 0.304). No hospital readmissions occurred during the postoperative period.

### 3.3. Functional Outcomes

At 6 months of follow-up, urinary continence rates were high in both groups, with no statistically significant differences between surgeons (Table 3). Erectile function recovery at 6 months, stratified according to the need for pharmacological or device-assisted support, was also comparable between groups, and no significant association with surgeon was observed. Rates of vesicourethral anastomotic stricture were low and did not differ between groups (Table 3).

### 3.4. Oncological Outcomes

Oncological outcomes are summarized in Table 4. The overall rate of positive surgical margins did not differ significantly between surgeon A and surgeon B, and among patients with margin positivity, the majority presented short margins (< 3 mm), with no significant differences in margin length distribution between groups.

BCR occurred in a minority of patients, with no significant differences observed between surgeons. PSA nadir values were comparable between groups, although a wide distribution was observed in both cohorts.

Pathological findings after radical prostatectomy were comparable between groups. Approximately 70% of patients presented organ-confined disease (pT2) (surgeon A: 40 vs. surgeon B: 30; *p* = 0.383), followed by locally advanced tumors (pT3a–pT3b), with no significant differences in pathological stage distribution between surgeons.

Similarly, definitive ISUP grade at prostatectomy did not differ between groups, with the majority of patients classified as ISUP grade 1–2 (surgeon A: 43 vs. surgeon B: 30; *p* = 0.608) and a low proportion of high-grade disease.

### 3.5. Pentafecta

Overall, pentafecta achievement was limited and comparable between surgeon A and surgeon B (23.6% vs. 21.1%, *p* = 0.77). As anticipated, pentafecta rates were lower than those observed for individual functional outcomes, reflecting the cumulative nature of this composite endpoint. Failure to achieve pentafecta was primarily driven by positive surgical margins, whereas functional recovery and postoperative morbidity were largely similar between groups.

**Figure 1:**
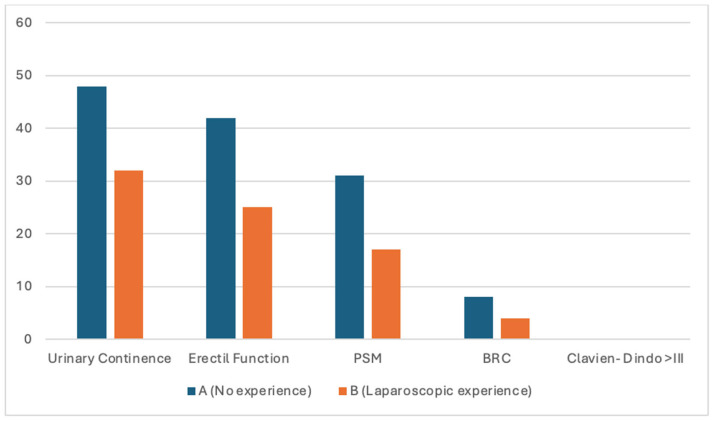
Distribution of individual pentafecta components by surgeon.

Exploratory analysis of the individual pentafecta (Figure 1) components showed high rates of urinary continence and erectile function recovery at 6 months (86% and 65%, respectively), with no major postoperative complications (Clavien–Dindo ≥ III). BCR was infrequent (12.9%). In contrast, negative surgical margins were obtained in less than half of the cohort (48.4%), emerging as the main limiting factor for pentafecta achievement. This pattern was consistent across both surgeons and may reflect, at least in part, a surgical strategy favoring functional preservation through systematic nerve-sparing, potentially at the expense of margin status in selected cases.

Bars represent the proportion of patients for each individual pentafecta component in each surgeon group. Pentafecta components include urinary continence, erectile function, positive surgical margins (PSM), biochemical recurrence (BCR), and Clavien–Dindo complications ≥ III.

Surgeon A had no prior laparoscopic experience before the robotic program, whereas Surgeon B had previous laparoscopic experience. This analysis is exploratory; formal hypothesis testing was not performed due to the low number of events for some outcomes.

## 4. Discussion

In this study, we evaluated perioperative, functional, and early oncological outcomes after robot-assisted radical prostatectomy performed by two experienced robotic surgeons with different surgical backgrounds after completion of the learning curve. Overall, early functional and oncological outcomes were similar between surgeons. These findings suggest that, beyond the initial learning phase, surgical performance may be more closely related to accumulated robotic experience and technical refinement than to prior laparoscopic training.

### 4.1. Surgical Performance and Operative Efficiency

Operative time was significantly shorter for surgeon A despite the absence of prior laparoscopic experience. Notably, prostate volume differed statistically between cohorts; however, the absolute difference in median volume was small and may be of limited clinical relevance. In contrast, differences in the distribution of prostate size (i.e., the presence of larger glands within the range) could still influence operative complexity and operative time, and this potential confounding is therefore acknowledged when interpreting these comparisons. This observation contrasts with previous reports indicating that laparoscopic background may facilitate early robotic adoption and shorten the initial learning curve. Studies by Prontera et al. [6] and Monnerat Lott et al. [9], among others, have shown that laparoscopic expertise is associated with faster attainment of operative efficiency during the early phase of robotic surgery.

Importantly, most available evidence focuses on the learning phase itself, whereas data addressing performance beyond the learning curve remain limited [11,13]. By restricting the analysis to procedures performed after both surgeons had surpassed a predefined learning threshold, our study provides insight into post-learning curve performance. In this context, differences in prostate volume should be considered when interpreting operative time comparisons. Nevertheless, the shorter operative time observed for surgeon A suggests that, once robotic proficiency is consolidated, factors such as repetition, workflow optimization, and cumulative surgical volume may play a more prominent role than prior laparoscopic experience. This aligns with previous observations indicating that the advantage conferred by laparoscopic training may diminish once robotic expertise is established [10,15].

### 4.2. Functional Outcomes and Surgeon Experience

Functional outcomes at 6 months, including urinary continence and erectile function recovery, were comparable between surgeons and consistent with rates reported in contemporary series [5,16,17]. These findings are consistent with population-based data indicating that surgeon experience and cumulative case volume are key determinants of functional recovery, rather than surgical background alone. Data from the Swedish registry, for example, have demonstrated progressive improvements in urinary continence with increasing surgeon volume following radical prostatectomy [17,18].

In the present study, both surgeons had completed the learning curve and applied standardized surgical techniques, which likely contributed to the homogeneity of functional outcomes. Systematic nerve-sparing was routinely pursued, prioritizing functional preservation. Although this approach may increase the risk of positive surgical margins in selected cases [19], it reflects a deliberate balance between oncological control and quality of life, particularly in patients with favorable-risk disease.

Rates of vesicourethral anastomotic stricture were low and comparable between surgeons, suggesting similar anastomotic quality and reconstruction technique after completion of the learning curve. The incidence observed is consistent with previously reported rates following RARP [20], further supporting the technical safety of standardized reconstruction when performed by experienced robotic surgeons.

### 4.3. Oncological Outcomes and Interpretation of Positive Margins

Oncological outcomes, including positive surgical margin rates, margin length distribution, biochemical recurrence, and PSA nadir, did not differ significantly between surgeons. The overall rate of positive surgical margins in our cohort was higher than that reported in some contemporary series and should be interpreted in the context of the systematic nerve-sparing strategy adopted to optimize functional outcomes within a balanced oncological–functional surgical approach [21,22]. Additionally, pathological assessment was based on a strict tumor-on-ink definition with standardized evaluation of margin involvement [23], which may have contributed to higher reported rates.

Importantly, most positive margins were short (< 3 mm), supporting the concept of heterogeneous oncological risk among patients with margin positivity [24,25,26]. The relatively short follow-up primarily captures early postoperative outcomes and may partly explain the low incidence of biochemical recurrence observed. In this regard, the limited number of recurrence events precludes definitive conclusions regarding long-term oncological equivalence between surgeons.

### 4.4. Pentafecta as an Exploratory Quality Metric

Pentafecta achievement was analyzed as an exploratory composite endpoint. In our cohort, overall pentafecta rates were modest and largely driven by the incidence of positive surgical margins, whereas functional recovery and perioperative safety outcomes were favorable. This pattern suggests that, in a post-learning curve setting, failure to achieve composite endpoints may primarily reflect oncological criteria rather than limitations in functional outcomes or surgical safety.

Although trifecta and pentafecta have been proposed as comprehensive quality metrics following radical prostatectomy [11,27,28], increasing evidence indicates that composite endpoints may oversimplify complex clinical trade-offs. Large contemporary series have shown that pentafecta achievement is strongly influenced by tumor risk profile rather than surgical technique alone. Bejrananda et al. [28], in a cohort exceeding 1600 patients, reported marked variability in pentafecta rates across risk groups, with positive surgical margins and biochemical recurrence emerging as the principal limiting components. Similarly, Afferi et al. [29] demonstrated that pentafecta achievement is highly dependent on risk stratification, underscoring that favorable functional outcomes may still be achieved even when composite endpoints are not fully met.

Within this framework, systematic nerve-sparing strategies may improve urinary continence and erectile function at the potential expense of margin status in selected patients, disproportionately affecting pentafecta rates [21,22]. These observations highlight the need for cautious interpretation of composite metrics and support the complementary evaluation of individual outcome components and tumor biology when assessing surgical quality [30]. Rather than invalidating the pentafecta as an outcome measure, the predominance of margin status in limiting its achievement in this cohort underscores its sensitivity to oncological factors and reinforces the importance of interpreting composite metrics in conjunction with individual outcome components.

### 4.5. Experience Versus Volume After the Learning Curve

The present findings contribute to the ongoing discussion regarding the relative roles of prior surgical background, learning curve completion, and cumulative surgical volume in RARP. While laparoscopic experience appears to facilitate early robotic adoption [6,9,10], our data suggest that, after completion of the learning curve, early outcomes tend to converge. In this setting, sustained surgical volume and progressive refinement of robotic skills may outweigh the influence of prior laparoscopic training. This aspect remains underexplored in the literature and represents a relevant contribution of the present analysis.

## 5. Limitations

This study has several limitations inherent to its retrospective, single-center design and the relatively short follow-up, which predominantly captures early oncological and functional outcomes. As a result, late biochemical recurrence and long-term functional recovery could not be fully assessed.

The study may be underpowered to detect small-to-moderate differences between surgeons, particularly for infrequent events such as biochemical recurrence and non-significant results may therefore reflect a Type II error rather than true equivalence between surgeons. In addition, pentafecta achievement was evaluated as an exploratory composite endpoint, intended to provide an integrated overview of surgical quality rather than to formally test a predefined hypothesis. In the small subgroup of patients with high-grade disease (ISUP 3–5), the limited sample size and lack of variability in nodal status precluded meaningful analysis of lymph node-related variables. Accordingly, lymph node management was not included in the statistical analysis, and no conclusions regarding its oncological impact can be drawn from the present study.

Nevertheless, the inclusion of consecutive patients, the use of standardized surgical techniques, and the restriction of the analysis to procedures performed after completion of the learning curve strengthen the internal validity of the study and allow for a focused assessment of post-learning curve performance.

## 6. Conclusions

In this exploratory post-learning curve analysis, early functional and oncological outcomes after robot-assisted radical prostatectomy were similar between two experienced robotic surgeons with different surgical backgrounds. These findings suggest that, once robotic proficiency has been achieved, early outcomes may be more closely related to cumulative robotic experience and surgical volume than to prior laparoscopic training.

Given the retrospective design, limited sample size, and short follow-up, these results should be interpreted with caution and considered hypothesis-generating. Further multicenter studies with longer follow-up are warranted to better characterize post-learning curve performance and its implications for surgical training pathways.

## Figures and Tables

**Table 1:** Baseline preoperative characteristics of patients undergoing robot-assisted radical prostatectomy according to surgeon.

	A (*n* = 55)	B (*n* = 38)	*p*
Age, years (median [IQR])	65 (46–74)	64 (49–76)	0.830
BMI, kg/m^2^ (median [IQR])	29.2 (28.5–29.9)	27.7 (27–28.4)	0.641
PSA, ng/mL (median [IQR])	6.76 (2.89–22.18)	7.09 (3.56–19.53)	0.105
PSA- Density ng/mL (median [IQR])	0.15 (0.06–0.64)	0.16 (0.07–0.59)	0.907
Prostate volume, mL (median [IQR])	43.0 (15–105)	46.0 (23–81)	0.025
MRI			0.076
PIRADs 3	7 (12.7)	11 (28.9)	
PIRADs 4	29 (52.7)	20 (52.6)	
PIRADs 5	19 (34.5)	7 (18.4)	
Biopsy ISUP grade, *n* (%)			0.758
1	24 (43.6)	19 (50.0)	
2	21 (38.2)	15 (39.5)	
3	6 (10.9)	2 (5.3)	
4–5	4 (7.3)	2 (5.3)	

Data are presented as median (interquartile range) or number (percentage), as appropriate. PSA: prostate-specific antigen; BMI: body mass index; MRI: multiparametric magnetic resonance imaging; PI-RADS: Prostate Imaging Reporting and Data System; ISUP: International Society of Urological Pathology.

**Table 2:** Intraoperative and postoperative outcomes according to surgeon.

	A	B	*p*
**Intraoperative outcomes**			
Operative time, min (median [IQR])	70 (60–100)	120 (80–165)	<0.001
Blood transfusion, *n* (%)	0	0	-
Conversion to open surgery, *n* (%)	0	0	-
**Postoperative outcomes**			
Clavien–Dindo complications			
Clavien–Dindo Grade I–II, *n* (%)	4 (7.3)	5 (13.2)	-
Clavien–Dindo Grade ≥ III, *n* (%)	0	0	-
Length of hospital stay, days (median [IQR])	1 (1–2)	1 (1–3)	0.304
Readmission, *n* (%)	0	0	-

Data are presented as median (IQR) or *n* (%), as appropriate. *p* values were calculated using the Mann–Whitney U test for continuous variables and Fisher’s exact test for categorical variables. NA indicates not applicable due to absence of events in both groups.

**Table 3:** Functional outcomes at 6 months according to surgeon.

	A	B	*p*
Urinary continence at 6 months, *n* (%)	48 (87.3)	32 (84.2)	0.77
Erectile function at 6 months (potent), *n* (%)	42 (76.4)	25 (65.8)	0.29
Vesicourethral anastomotic stricture, *n* (%)	2 (3.6)	1 (2.6)	1.00

Data are presented as *n* (%). *p* values were calculated using Fisher’s exact test. Potency was defined as the ability to achieve erections sufficient for intercourse with or without pharmacological support.

**Table 4:** Oncological outcomes according to surgeon.

Outcome	A	B	*p*
Positive surgical margin, *n* (%)	31 (56.4)	17 (44.7)	0.270
Margin length > 3 mm, *n* (%)	9 (29.0)	5 (29.4)	0.978
Biochemical recurrence, *n* (%)	8 (14.5)	4 (10.5)	0.570
PSA nadir, ng/mL (median [IQR])	0.02 (0.00–0.03)	0.02 (0.00–0.03)	0.759

Data are presented as median (IQR) or *n* (%). *p* values were calculated using the Mann–Whitney U test or Fisher’s exact test, as appropriate.

## Data Availability

The data presented in this study are not publicly available due to ethical and privacy restrictions related to patient confidentiality. Data are available from the corresponding author upon reasonable request.

## Abbreviations

The following abbreviations are used in this manuscript:

BCR	Biochemical recurrence
ISUP	International Society of Urological Pathology
PSA	Prostate-specific antigen
PI-RADS	Prostate Imaging Reporting and Data System
RARP	Robot-assisted radical prostatectomy
